# MgCuSb as a suitable electrode for contacting pre-compacted pellets of MgAgSb thermoelectric material

**DOI:** 10.1080/14686996.2025.2506982

**Published:** 2025-05-27

**Authors:** Amandine Helt, Amandine Duparchy, Aidan Cowley, Eckhard Müller, Johannes de Boor

**Affiliations:** a Institute of Materials Research, German Aerospace Center (DLR), Cologne, Germany; b European Astronaut Centre, European Space Agency (ESA), Cologne, Germany; c Institute of Inorganic and Analytical Chemistry, Justus Liebig University of Giessen, Giessen, Germany; d Institute of Technology for Nanostructures (NST) and CENIDE, Faculty of Engineering, University of Duisburg-Essen, Duisburg, Germany

**Keywords:** MgAgSb, thermoelectricity, devices, contacting, MgCuSb, electrode, interface, contact resistances, microstructure

## Abstract

The p-type thermoelectric (TE) material α-MgAgSb is a promising tellurium-free bismuth telluride substitute for cooling and waste heat harvesting applications between room temperature and 573 K. Optimization of the material resulted in high values of figure of merit (*zT*_max_ = 1.3) to date, but performance optimization of TE devices also requires minimizing the electrical contact resistance between the TE material and the electrodes. Here, we investigate the metallization of MgAgSb with MgCuSb, providing microstructural and electrical analyses of the interfaces for functionalized legs obtained from a combined sintering of both materials systematically varying temperature, duration and pressure. Analysis of the obtained results reveals the formation of an interdiffusion layer of Ag_3_Sb with varying thickness in all samples, but the contact resistance remains consistently below 10 μΩ cm^2^. Microprobe measurements of the Seebeck coefficient indicate a change in carrier concentration in the TE material close to the interface, visualizing interdiffusion processes between MgAgSb and MgCuSb. We furthermore demonstrate that MgCuSb can successfully be applied as an electrode on pre-compacted MgAgSb samples, resulting in the first ever reported successful two-step contacting of MgAgSb. The obtained sample exhibits a strong mechanical contact without any crack at the interface, as well as a very low electrical contact resistance below 7 µΩ cm^2^, representing less than 5% of the total leg resistance. Successful contacting of pre-compacted material is a step forward towards module fabrication as it enables better control of the TE leg length and thus device performance.

## IMPACT STATEMENT

This paper presents MgCuSb as a suitable electrode for MgAgSb thermoelectric material using microstructural and electrical analyses, as well as the first successful contacting of MgAgSb on pre-sintered pellets.

## GRAPHICAL ABSTRACT

**Figure:**
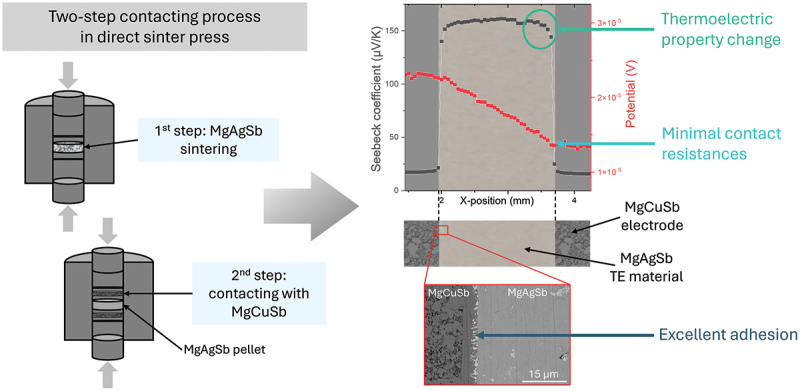

## Introduction

In the current context of energy crisis and climate change, one of the biggest challenges is to produce enough electricity to meet the growing demand while trying to have the most environmentally friendly energy possible [1,2]. In this regard, a step forward to the future of clean energy production and harvesting would be the use of devices based on thermoelectric (TE) effects. On the one hand, thermoelectric coolers (TECs) using the Peltier effect would help reducing the emission of fluorinated refrigeration gases, which represent a substantial amount of the total greenhouse gas emissions [3,4]. On the other hand, thermoelectric generators (TEGs), that are capable of converting a temperature difference into electricity using the Seebeck effect, are promising devices in the field of energy harvesting [5,6] for recovering waste heat, e.g. in cars, homes and various industrial processes [7]. Another area of application for TEGs is the aerospace industry, where Radioisotope Thermoelectric Generators (RTGs) have already been used to power the Voyager probes, Cassini to Saturn or Mars rovers like Curiosity and Perseverance [8,9], mainly because they are lightweight, scalable and reliable, requiring no maintenance as they do not have any moving parts [10]. For future lunar missions like Artemis, those devices will play a key role to provide electricity when sunlight is not available [11].

A thermoelectric generator is composed of p- and n-type thermoelectric legs functionalized on two sides by metallic electrodes. These electrodes aim to facilitate the soldering between the legs and the copper bridges that connect the legs electrically. The performance of a TEG is scaled by its maximum power output, *P*_el,max_, as well as by the maximum conversion efficiency *ɳ*_max,_ as given for a single leg by the following equations [12]:

(1) $$P_{\mathrm{el},\max}=\frac{\alpha^{2}\sigma }{2}\frac{A{\left(T_{\mathrm{hot}}-T_{\mathrm{cold}}\right)}^{2}}{\left(L+m\right){\left(1+2\mathrm{rw}\right)}^{2}}$$

(2) $$\eta_{\max}=\frac{T_{\mathrm{hot}}-T_{\mathrm{cold}}}{T_{\mathrm{hot}}}\text{\textbackslash{}break}{\left({\left(1+2\mathrm{rw}\right)}^{2}\left[2-\frac{1}{2}\frac{T_{\mathrm{hot}}-T_{\mathrm{cold}}}{T_{\mathrm{hot}}}+\frac{4}{zT_{\mathrm{hot}}}\left(\frac{L+m}{1+2\mathrm{rw}}\right)\right]\right)}^{-1}$$

$T_{\mathrm{hot}}$ and $T_{\mathrm{cold}}$ are the temperatures at the hot and cold sides of the element, respectively; *L* and *A* are the length and the cross-sectional area of the leg, respectively; and r =κ /κ_c_, *w* = *L*_c_/*L*, *m* = 2*r*_c_/ρ, where the subscript c indicates the properties of the contacts. Note that *r*_c_ here is the specific contact resistance for a single contact (Ω m^2^), ρ is the resistivity of the leg (Ω m), and thus *m* has the unit of length.

Both parameters largely depend on the TE material properties, the geometry of the leg and the contact resistances between the TE leg and the electrodes. The material properties are summarized by the thermoelectric figure of merit $\mathrm{zT}=\frac{\alpha^{2}\sigma }{\kappa }T$, with *α* the Seebeck coefficient, *σ* the electrical conductivity, *κ* the thermal conductivity and *T* the absolute temperature [13].

While the functionalization of the TE legs is crucial to facilitate their soldering to the copper bridges, it should also protect the TE material from copper diffusion [14], provide a transition in the coefficient of thermal expansion (CTE) [15] and cause minimal electrical and thermal contact resistances at the interface [15]. That is why it is essential to find an electrode that keeps the contact resistance low, prevents delamination, limits interfacial reactions and a change in carrier concentration due to atomic interdiffusion [16], all under the effect of mechanical and thermal stresses [17]. Finally, a process with parameters that do not degrade the TE material during the joining needs to be established. From a technological perspective, it is also advantageous to apply the electrode on pre-compacted legs (known as two-step joining process) to enable precise adjustment of the height, rather than in a single combined compaction step. Indeed, with the latter, it is more challenging to obtain precise and homogenous TE height due to, e.g. powders mixing at the interface or variations in density.

In the past decades, significant progress has been made in the thermoelectric field with the development of highly efficient TE materials (*zT* > 1) such as Bi_2_Te_3_ [18–21], skutterudites [22–25] or Half-Heusler [26–29]. Mg-based TE materials like Mg_3_Sb_2_ [30,31], Mg_2_*X* (*X* = Si, Sn) [32,33] or MgAgSb [34,35] have been more recently identified as promising substitutes for Bi_2_Te_3_ in the range of low to mid temperatures (i.e. from room temperature to around 575 K) for waste heat recovery or cooling applications [18]. Indeed, modules made of these materials have shown comparable or higher efficiencies [36–38] than those obtained with the state-of-the-art, while being composed of globally more abundant, cheaper and less toxic elements. Devices using α-MgAgSb as the p-type material show, for instance, a maximum conversion efficiency of 6.4% and a maximum power output of 1.03 W cm^−2^ (normalized to the TE material area) with Mg_2_(Si,Sn) solid solution as the n-type counterpart [39], or even a maximum conversion efficiency of 8.5% when combined with Mg_3_Sb_2_ [40]. While those works demonstrated remarkable progress, they also pointed out that electrical contact resistances are responsible for power and efficiency losses up to 20% [39].

This study therefore focuses on the functionalization of the p-type thermoelectric material MgAgSb (α-phase), which was first identified by Kirkham et al. [41]. The synthesis process has then been optimized in a number of recent works [34, 42–44] to reach a maximum *zT* of around 1.3 at 575 K with a nominal composition of MgAg_0.97_Sb_0.995_ [34]. So far, mainly due to their close CTEs (19.5 × 10^−6^ K^−1^ and 20 × 10^−6^ K^−1^ respectively for Ag and MgAgSb [34]), its ductility and its high electrical conductivity (6.30 × 10^7^ S m^−1^ at 295 K [45]), Ag has been mostly used as an electrode for MgAgSb using a one-sintering-step contacting process and showed successful results with good performance and suitability [35, 39, 40, 46, 47]. However, conflicting data exist as it has also been shown that the contact resistance of the Ag/MgAgSb interface is not stable over time, leading to the formation of cracks [48]. It can be noted that no successful attempt of the functionalization of MgAgSb with Ag using a two-step sintering process is reported in the literature, which is probably due to the too high melting temperature of Ag. Indeed, on the one hand, the joining temperature of α-MgAgSb TE material should not exceed 625 K to avoid the formation of the detrimental and metastable γ-phase of MgAgSb [41, 49]. On the other hand, based on own tests [50] and general joining guidelines stating that the sintering temperature should be higher than 0.4 times the melting temperature of the electrode (both in °C) [48, 51], we find that the maximum acceptable melting temperature of a potential electrode for the functionalization of α-MgAgSb via a two-step process is 1150 K (below the melting temperature of Ag being 1235 K [52]). That is why it is crucial to find a new electrode for MgAgSb, suitable with a two-step contacting process and leading to a stable interface.

In a recent publication, Xie et al. [48] developed a screening strategy for thermoelectric interface materials and presented the semi-metal MgCuSb as a promising electrode for MgAgSb. Out of 17 candidates that were selected because they have a two-phase equilibrium with MgAgSb (which should prevent the formation of detrimental secondary phases at the interface), MgCuSb was the one with the lowest electrical resistivity for a melting temperature below 1150 K that could be successfully synthesized. An ultra-low contact resistivity of less than 1 μΩ cm^2^ for the TE material/electrode interface was demonstrated, maintained also after a 2-week annealing, as well as a conversion efficiency of 9.25% with a two-pair module using Mg_3.2_Bi_1.5_Sb_0.5_ as the n-type counterpart in a temperature difference of 575 K. However, it is important to note that the p-type TE material used in these two experiments is not MgAgSb single phase but a composite in which they substituted 10% of Ag by Cu, leading to a nominal composition of MgAg_0.87_Cu_0.1_Sb_0.99_. This affects the functional properties of the TE legs as well as the joining process and the resulting interface properties.

In this work, we therefore investigate for the first time the contacting of α-MgAgSb with MgCuSb, as the theoretical screening strategy presented by Xie et al. [48] pointed out the suitability of this material combination. Several samples were successfully contacted via a one-step process, using different sintering temperature, duration and pressure. The effect of these parameters on the contact quality is examined by microstructural and electrical analyses. We show that, even if increasing the joining temperature and duration leads to the formation of a larger interdiffusion layer, it also decreases the electrical contact resistance, indicating a better electrical contact between the TE material and the electrode. Following the promising results obtained for one-step contacted samples, we demonstrate the first successful two-step contacting of MgAgSb. The analysis of the junction shows a good adhesion of the two materials without any visible cracks, and a low electrical contact resistance of less than 5% of the total leg resistance for a typical leg geometry is obtained.

## Materials and methods

The powder of the p-type thermoelectric material α-MgAgSb was synthesized with a nominal stoichiometry of MgAg_0.97_Sb_0.995_ following the route described by Duparchy et al. [44]. It consists of an initial ball milling step of Mg with Ag for 8 h, followed by an 8-min sintering at 675 K under 85 MPa which aims to ensure the formation of the MgAg phase. The milling jar is then cleaned to get rid of possible Mg and Ag leftovers that could initiate the formation of secondary phases if they react with Sb. Then, the pellet is ground, weighed and crushed before the addition of Sb in stoichiometric quantities, and a second ball milling step of 5 h is performed.

For the synthesis of the MgCuSb electrode, the route described by Xie et al. [48] was initially used. Stoichiometric amounts of Mg flakes (turnings, Merck KGaA, >99%), Cu powder (Nuclear Metals Inc., 99.95% purity) and Sb chunks (Sindlhauser Materials GmbH, 99.99% purity) were weighed according to the composition Mg_1_Cu_1_Sb_1_ and put in a stainless-steel jar (65 ml) with two stainless steel balls (12.7 mm diameter) under argon atmosphere. High-energy ball milling (SPEX SamplePrep 8000D Mixer/Mill) was then performed for 20 h, with hammering steps every 3 h to remove the powder stuck on the jar walls and ensure the complete reaction of the elements to form MgCuSb. To study the quality of the synthesized material, milled MgCuSb powder was sintered for 30 min at 575 K under 85 MPa and studied by X-ray diffraction using a Bruker D8 diffractometer with a secondary monochromator, Co-Kα radiation (1.78897 Å) and a step size of 0.019° in the 2*θ* range of 20–80°. As the refinement of the obtained pattern (Figure S1, red pattern) indicates the presence of Cu_2_Sb in an amount up to 11 wt.% in the MgCuSb matrix (Table S1), a second synthesis route based on that of MgAgSb and consisting in two ball milling steps of 10 h each was evaluated. This synthesis route ensures the complete reaction of magnesium and copper through a sintering step performed for 10 min at 675 K after the first ball milling step, before adding antimony according to the Mg_1_Cu_1_Sb_1_ composition. The powder was then sintered and studied with XRD (Figure S1, black pattern), showing a content of 6 wt.% Cu_2_Sb (Table S2). In contrast to MgAgSb synthesis, for which the intermediate precursor is MgAg, the stable phases obtained when mixing 50 at.% Mg and 50 at.% Cu at 575 K are Mg_2_Cu and MgCu_2_ [53]. This will lead to local deficiencies/excess of Mg and/or Cu, which would lead to Cu-rich/poor phases when adding Sb, and thus explains the challenge to obtain secondary-phase-free MgCuSb by this route, while it is possible for MgAgSb.

The microstructure of both obtained pellets was studied using a Hitachi High Tech’s SU3900 Scanning Electron Microscope (SEM) and can be found in Figure S2. Both images show a porous material, which makes it difficult to see secondary phases or any other difference between both samples. Some of their properties are presented in Table S3 and show similar values. In addition, as the powders obtained from these two syntheses did not lead to significant differences regarding the interface properties when being used for MgAgSb functionalization, they are discussed equally in the following.

**Figure 1.**
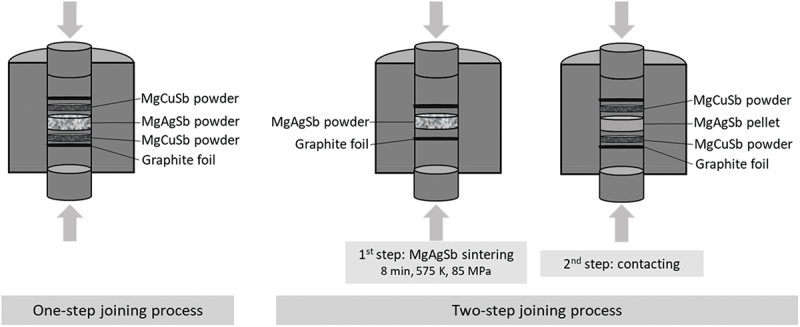
One- and two-step joining processes. Graphite foils are used to facilitate the separation of the sample from the pressing stamps after sintering.

Both TE material and electrode powders were loaded in a layered stacking (see Figure 1) into 12.7-mm diameter graphite dies to fabricate one- and two-step contacted pellets using a direct sintering press (Dr Fritsch DSP510). The one-step process consists of hot pressing three stacked layers of MgCuSb/MgAgSb/MgCuSb in the powder form. For the two-step process, MgAgSb powder is first hot-pressed independently, and the resulting pellet is then loaded into the die with MgCuSb powder layers on top and bottom for a second hot pressing step, after adjusting the TE length by grinding. Several parameter sets for sintering time, temperature and pressure have been employed, as reported in Table 1, and are represented in the sample name whose form is: Process _Temperature (K) – Duration (min) – Pressure (MPa)_. All sintering was conducted under vacuum to avoid oxidation.

**Table 1.** Sintering parameters used for the contacting experiments.

Sample name Process _K-min-MPa_	Temperature (K)	Time (min)	Pressure (MPa)
1step_575-30-85_	575	30	85
1step_575-8-85_	575	8	85
1step_625-8-85_	625	8	85
1step_575-30-200_	575	30	200^a^
2step_575-30-85_	575	30	85

For the two-step hot pressing, the parameters given in the table are those used for the contacting step, while for MgAgSb compaction, 575 K, 30 min and 85 MPa were employed.

^a^This sample has been hot pressed in a stainless-steel die, instead of a graphite die as the latter cannot support more than 100 MPa.

After the joining step, the pellets were cut into bars and the microstructure of the cross-sectional surfaces was observed using SEM and Energy Dispersive X-ray (EDX) spectroscopy. The quality of the joining between the electrode and the TE material was then gauged quantitatively using an in-house built device called Potential & Seebeck Microprobe (PSM) [54, 55]. This device measures locally the surface Seebeck coefficient and the electrical potential over the cross-section of the sample with a here employed spacing of 0.05 and 0.15 mm between the points in a line scan and the line scans, respectively. The voltage drop across the sample is measured using a lock-in amplifier, while the current is determined by measuring the voltage over a 1 Ω precision shunt resistor, connected in series with the Cu blocks of the sample holder. The uncertainty of the Seebeck coefficient measurement is reported to lie between 10% and 15% due to the cold finger effect [55]. By evaluating the electrical potential scan, it is possible to determine the specific electrical contact resistance *r*_c_ (μΩ cm^2^). It can be calculated using Equation (3), where *V*_elec_ *- V*_TE_ is the voltage drop measured using linear fits of the potential profile, within the electrode and the TE material respectively, extrapolated to the interface position which is determined using the drop in Seebeck coefficient (see Figure S3). *A* is the studied cross-sectional area of the sample and *I*_PSM_ is the AC current value [56,57].

(3) $$r_{c}=\frac{\left(V_{\mathrm{elec}}-V_{\mathrm{TE}}\right)*A}{I_{\mathrm{PSM}}}$$

This equation is strictly valid only if the current density is homogeneous throughout the sample. The results presented in this work correspond to the average *r*_c_ values of all valid line scans measured for each sample, and were calculated using a MATLAB program specifically designed for this purpose, as described in Figure S3.

It is not possible to define a general target value of *r*_c_ to assess the quality of a joining as this is a function of the conductivity and the length of the employed TE material. Here, the TE length is around 2 mm which is typical for thermoelectric modules. The relative contact resistance parameter *n* (Equation (4)) can thus be used to compare the global contact quality of the different samples, and is representative of this application case:

(4) $$n=\frac{2R_{c}}{2R_{c}+R_{\mathrm{TE}}}$$

In this equation, *R*_c_ is the contact resistance on one side and *R*_TE_ the internal resistance of the TE material. The contact resistance is here multiplied by two in order to consider the two sides of the contacted sample in the same expression and have only one value to compare per sample. The joining will be considered as favorable for *n* < 15%, as it has been shown in paper [58] that this is the maximum value to avoid reducing the conversion efficiency by more than 10%.

## Results

To check the compatibility of MgCuSb as an electrode for MgAgSb and determine the best sintering parameters with respect to adhesion and electrical contact resistance, several one-step contacting experiments were performed using the parameters reported in Table 1.

**Figure 2.**
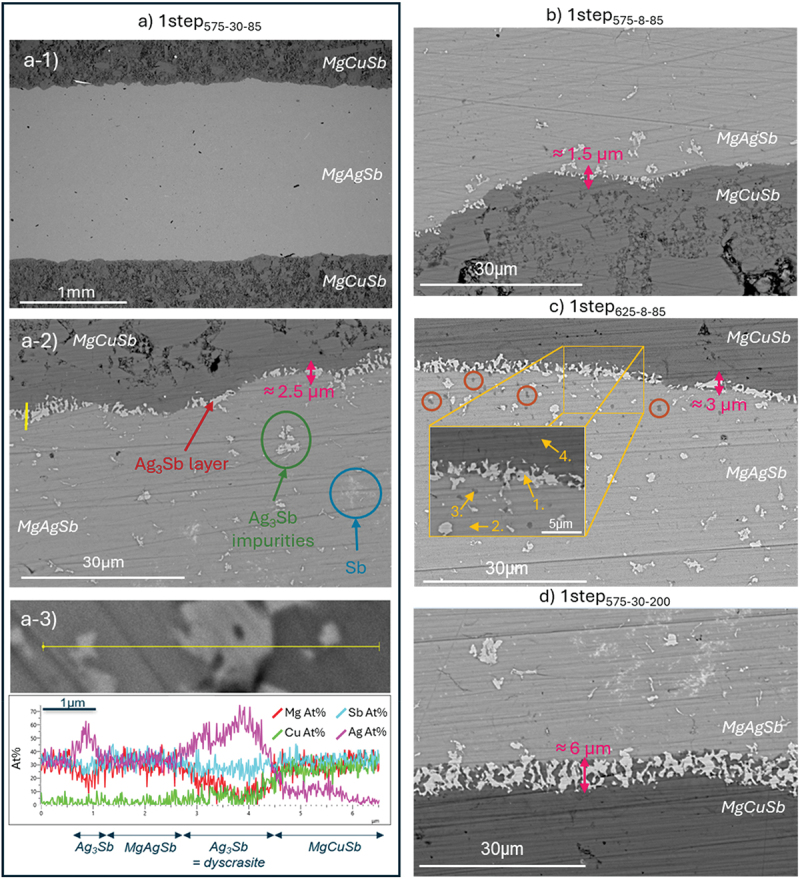
Backscattered electron (BSE)-SEM images of the one-step contacted samples. (a) corresponds to 1step_575-30-85_ with (a-1) exemplary low magnification BSE-SEM image of the cross section, typical for all one-step samples; (a-2) zoom on the interface showing different secondary phases, with the yellow line corresponding to the EDX line-scan location shown in (a-3). (b), (c) and (d) are BSE-SEM images of the interfaces of 1step_575-8-85_, 1step_625-8-85_ and 1step_575-30-200_, respectively. In (c), the numbers in the zoomed-in section correspond to the points of the EDX analysis, given in Table 2, and the brown circles highlight areas in which copper has been detected. The thickness of the Ag_3_Sb layers is indicated by a pink arrow and was calculated by averaging values measured at different points along the interface.

The SEM/EDX analysis of the MgCuSb/MgAgSb interfaces is displayed in Figure 2.

**Table 2.** Composition of the MgCuSb/MgAgSb interface for sample 1step_625-8-85_ obtained using EDX measurement on points 1–4 as indicated in Figure 2c.

Point	Mg (at. %)	Ag (at. %)	Sb (at. %)	Cu (at. %)	Phase
1.	3.8	24.8	69.6	1.8	Ag_3_Sb
2.	32.3	33.4	34.4	/	MgAgSb
3.	32.1	29.3	32.4	6.2	Mg + Ag + Sb + Cu
4.	34.0	1.6	32.4	29.6	MgCuSb

**Figure 3.**
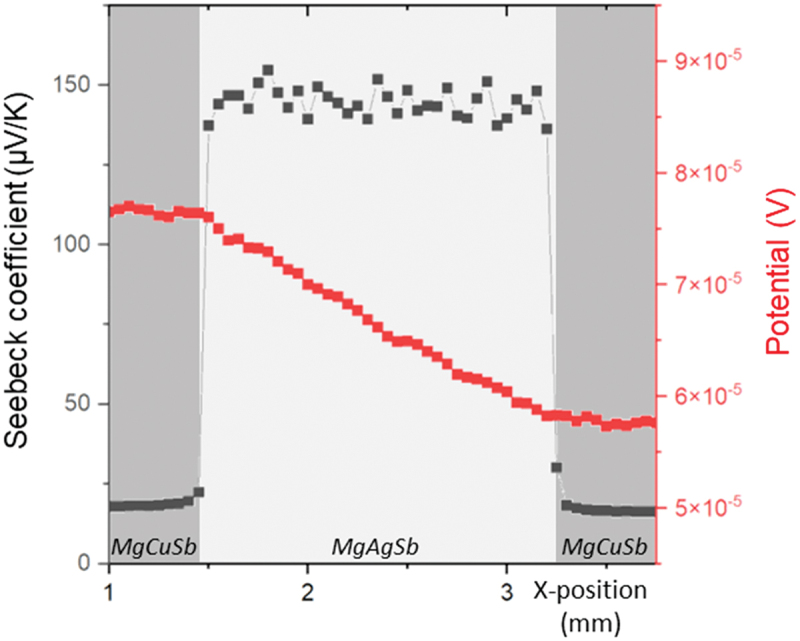
Line scan of the Seebeck coefficient and the electrical potential over the cross section of sample 1step_575-30-85_.

The low magnification view of the one-step contact cross section is given for sample 1step_575-30-85_ in Figure 2(a-1). As the cross sections of the other three samples look similar (see Figure S4), only one is presented. Figure 2(a-1) shows a good adhesion of the two materials as no delamination or cracks can be seen. Nevertheless, light grey phases can be observed at the interface for all the samples, mainly on the electrode side, as indicated by the red arrow in Figure 2(a-2). From the EDX analyses performed on sample 1step_575-30-85_ (Figure 2(a-3)) and sample 1step_625-8-85_ (point 1, Figure 2c and Table 2), these phases can be identified as dyscrasite (Ag_3_Sb). Depending on the samples, the reaction zones where dyscrasite is formed can have different thicknesses as highlighted by the pink arrows in Figure 2. For each sample, the given thickness is an average of several values measured along the interface.

Farther into the TE material, Ag_3_Sb (white impurities, as highlighted by the green circle in Figure 2(a-2)) and Sb (white blurry phases, see blue circle in Figure 2(a-2)) are also present. Such phases probably originate from the synthesis of the TE material itself as they can also be observed in non-contacted samples (Figure S5). It is well established that secondary phases easily form in MgAgSb [43,44], mainly due to powder sticking on the ball milling jar walls leading to difficulties in controlling stoichiometry. Solutions to overcome this issue would be the use of control agents such as stearic acid, as demonstrated in paper [59], or the control of MgAg stoichiometry to avoid local fluctuations [60]. However, Ag_3_Sb impurities seem here to be more abundant close to the interface than farther away (Figure 2b), meaning that one cannot exclude the possibility that some Ag_3_Sb phases might also form due to some reaction between MgAgSb TE material and MgCuSb electrode.

On sample 1step_625-8-85_, black spots are visible in the TE material close to the interface (Figure 2c, brown circles). The EDX point analysis presented in Table 2, point 3, reveals that they are composed of Mg, Ag and Sb, elements expected as in the MgAgSb matrix, but also of 6.2 at.% of Cu. The electrode, which is the only possible source of copper, contains less copper than expected according to what was measured for a non-contacted MgCuSb sample (Figure S6), further indicating diffusion of Cu into the TE material (Table 2, point 4). Moreover, when comparing the compositions of points 2 and 3 (Table 2), it is found that Cu is localized in the dark areas, as no Cu is detected in point 2. Due to resolution limitations, it cannot be concluded if these dark spots are single phase, but it has been reported that the solubility of Cu in MgAgSb is limited to much smaller values than measured here [61], indicating a phase mixture.

In Figures 2d and S4-c, one can see that the MgCuSb electrode for 1step_575-30-200_ contacted under 200 MPa is much less porous than for the other samples whose contacting pressure was 85 MPa (Figures 2a-c and S4-a&b).

To investigate further the effect of the sintering parameters on the interface quality, measurements of electrical potential and Seebeck coefficient were performed using the PSM.

An exemplary line scan measured along the cross-section is displayed in Figure 3 for sample 1step_575-30-85_. The scans for the other samples can be found in Figure S7 and look similar. It can be noticed that the potential curves are linear in the electrode and over the TE material, which is an indication of a good homogeneity of both materials with respect to electrical conductivity and current density, meaning that Equation (3) can be used to calculate the electrical specific contact resistance values in good approximation. The obtained values are presented in Table 3 for all one-step contacted samples, as well as the relative contact resistance parameter *n* (Equation (4)) which shows that the contact resistance contributes to less than 5% of the total resistance of the bar, assessing a good interface quality [58]. The low contact resistance values are in agreement with the absence of visible potential drops at the interface between the electrode and the TE material, as well as with the fact that the contacts are Ohmic contacts, as indicated by the larger work function for MgCuSb (4.68 eV) than for MgAgSb (4.56 eV) determined by first principles calculations [48,62]. However, one can notice that some of the contact resistance values are obtained as negative values, which is physically not possible but can be explained by the excellent electrical properties of the contacts, in combination with significant noise (as evidenced by the fact that the standard deviation is of the same magnitude as the resistance value itself), and potentially a systematic over- or underestimation if the potential profile is not perfectly linear but slightly bent (see Figure S3 for more information on the fitting procedure used for the calculation). It should also be noted that the spacing between the PSM measurement points is 50 µm, which is significantly larger than the interdiffusion zones visible in Figure 2. The obtained contact resistances thus comprise actual interface resistances as well as potential bulk resistances of the formed phases.

**Table 3.** Specific electrical contact resistance values, relative contact resistance parameter n (see Equation (4)) and thicknesses of the dyscrasite layer for all the MgAgSb samples contacted with MgCuSb using a one-step process.

	*r*_c_ (µΩ cm^2^)	n (%)	Thickness of the Ag_3_Sb layer (μm)
**1step**_**575-30-85**_	0.7 ± 5	0.2 ± 2	2.5
**1step**_**575-8-85**_	6 ± 10	2 ± 3	1.5
**1step**_**625-8-85**_	0.9 ± 3	0.3 ± 0.9	3
**1step**_**575-30-200**_	−2 ± 3	−0.7 ± 1	6

For each sample, the given *r*_c_ value is an average of the values calculated with Equation (3) for all valid line scans, left and right.

Contacting MgAgSb with an MgCuSb electrode at 575 K for 30 min results in a lower contact resistance than for the 8-min contacted sample. Moreover, for 8 min of sintering, a temperature of 625 K leads to a lower contact resistance than 575 K. It thus seems that both increasing the sintering time and the sintering temperature enables to reduce the contact resistance. In addition, the same observation can be made regarding the standard deviation values. However, when looking at the thickness of the dyscrasite layer, one can notice that it increases with both the sintering time and temperature, meaning that it is negatively correlated to the contact resistance. Regarding the comparison of samples 1step_575-30-85_ and 1step_575-30-200_, it seems that increasing the sintering pressure leads to the formation of a larger Ag_3_Sb layer, but no clear link with the contact resistance can be done. It should be noted, however, that the values for all samples are quite small and the uncertainties are comparable to the measurement values, so conclusions must be drawn carefully.

The sintering parameters used for the contacting step of the sample 2step_575-30-85_ were chosen the same as those of 1step_575-30-85_ since this sample did not show any Cu diffusion, unlike 1step_625-30-85_, and also exhibited a lower contact resistance than 1step_575-8-85_ and a thinner dyscrasite interface zone than 1step_575-30-200_.

**Figure 4.**
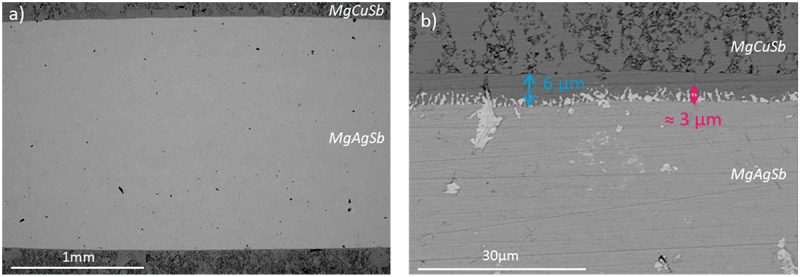
BSE-SEM images of 2step_575-30-85_ with (a) low magnification image of the cross section, and (b) zoom near the top interface. The thickness of the Ag_3_Sb interface zone is indicated by a pink arrow and was calculated by averaging values measured at different points along the interface. The blue arrow shows the thickness of a homogeneous MgCuSb layer along the interface.

The SEM analysis of the sample is presented in Figure 4. Similarly to what has been observed for 1step_575-30-85_, the two-step contacted sample does not show any delamination or crack at the MgAgSb/MgCuSb interface (Figure 4a). The same dyscrasite interface zone can be seen, still mainly located on the electrode side, however a bit thicker with a width of around 3 μm, and more homogeneously spread along the interface (Figure 4b). Further differences between the two samples are the flatter interface, which is due to the process as the electrode has been applied on a pre-sintered pellet with a flat base face, and the 6-μm-thick MgCuSb compacted layer present along the interface and highlighted by a blue arrow in Figure 4b. A closer view of the interface as well as an EDX point analysis of the observed phases can be found in Figure S8.

**Figure 5.**
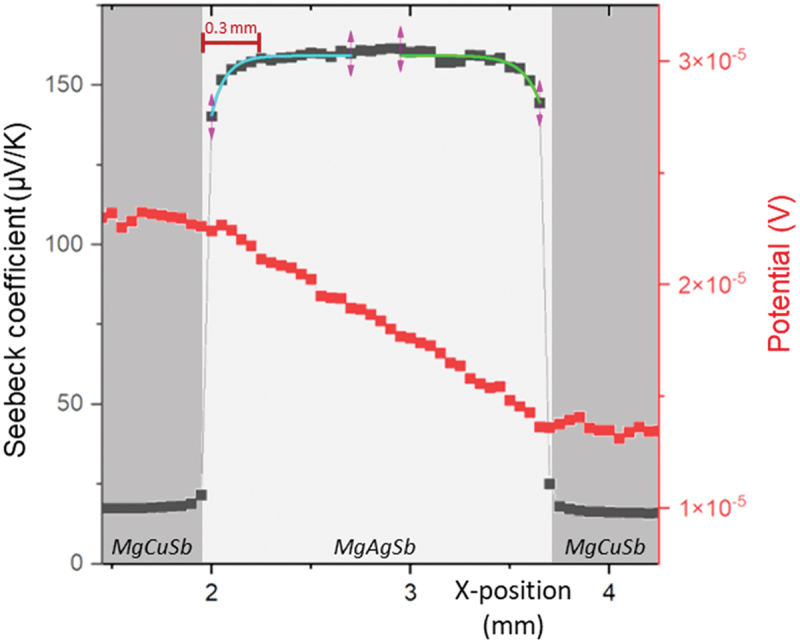
PSM line scan of the Seebeck coefficient and the electrical potential for the cross section of 2step_575-30-85_ sample.

The PSM line scan of 2step_575-30-85_ is presented in Figure 5. Similarly to that of 1step_575-30-85_, the potential curves are roughly linear in the electrodes and the TE material. In addition, no large potential drop can be seen at the interface between the two materials, consistent with the low contact resistance value standing below 7 µΩ cm^2^. The calculated *n* ratio (Equation (*4*)) is meanwhile lower than 4%, which assesses a good interface quality [58]. Both values are similar within the measurement uncertainty to those obtained for 1step_575-30-85_. However, it can be noticed from comparing the PSM line scans of 1step_575-30-85_ (Figure 3) and 2step_575-30-85_ (Figure 5), that the Seebeck coefficient in the MgAgSb section is less scattered in the second case, and that the average Seebeck coefficient value increased from around 145 to 160 μV K^−1^ between the two samples. Those changes are due to the fact that the second step of the contacting route acted like an additional annealing treatment on the TE material, leading to the transformation of β-MgAgSb (remnant from the material sintering step) into the desired α-MgAgSb, resulting in a functionally more homogeneous material with better TE properties, as evidenced by the constant and higher Seebeck values. Another noticeable difference between Figures 3 and 5 is the Seebeck coefficient gradient visible on both sides of the TE material, close to the interface, in the PSM scan of 2step_575-30-85_. To quantify this gradient, an exponential fitting of the curve has been employed using $S\left(x\right)=S_{\mathrm{bulk}}+A\exp\left(\frac{x_{0}-x}{\tau }\right)$ on the left side and $S=S_{\mathrm{bulk}}+A\exp\left(\frac{x-x_{0}}{\tau }\right)$ on the right side (respectively blue and green curves in Figure 5) [17]. In these equations, *x*_0_ is the first point in the TE material close to the interface, *A* gives the difference of the Seebeck coefficient between the undisturbed TE material $S_{\mathrm{bulk}}$ and the value at the interface, *τ* is the diffusion length and *S* is the Seebeck coefficient at the position *x*, expressed in μV K^−1^. The diffusion length represents the distance from *x*_0_ at which the difference in Seebeck coefficient has reduced to 1/e^1^ = 37% of its maximum value.

After fitting all the valid PSM line scans for this sample, the averages of both parameters *τ* and *A* are calculated and result in a diffusion length of around 90 µm, as well as a difference of 20 μV K^−1^ between the Seebeck coefficient in the middle of the TE material and at the interface, which is a clear hint to a change in the TE properties, probably due to a change of carrier density.

## Discussion

The combination of the SEM (Figure 2) and PSM (Figure 3 and Table 3) analyses for one-step contacted samples enables to draw conclusions about the optimum contacting parameters. Firstly, comparing results for 1step_575-8-85_ and 1step_575-30-85_ points out that, while increasing the sintering duration enables the dyscrasite interface zone to grow more by allowing longer diffusion, it also leads to a reduction of the electrical contact resistance and its standard deviation. The latter effect is certainly due to the stronger interdiffusion at the interface, enabled by the longer hot-pressing step and leading to a stronger bonding between the electrode and the TE material.

Secondly, increasing the contacting temperature can lead to better contacts as the materials are closer to their melting point and thus interdiffusion and local reactions are increased [63–65]. This is verified in this study as 1step_625-8-85_ shows a lower contact resistance (with a lower standard deviation value) than 1step_575-8-85,_ for the same sintering duration. However, a higher temperature also enhances diffusion, not only at the very interface as it can be seen with the thicker Ag_3_Sb interface zone compared to 1step_575-8-85_ (Figure 2b,c) but also deeper into the TE material, as visible from the Cu-rich regions observed in 1step_625-8-85_ (Figure 2c, brown circles). This last phenomenon is to be avoided as it would lead to a modification of the TE material composition and thus of the TE properties, which would affect the performance of a module. Furthermore, as contacting was performed at 625 K, β-MgAgSb forms instead of the desired α-phase, influencing the properties of the TE material [41,66]. The β-phase can be converted into the α-phase quickly by annealing just below the phase transition temperature of 605 K [41], which is needed for instance when controlling the quality of the material or device, but this would require the introduction of an additional annealing step, and thus lead to a longer process. As the contact resistances and the thicknesses of the dyscrasite interface zones of 1step_575-30-85_ and 1step_625-8-85_ are similar within the measurement uncertainty, one can conclude that it is preferable to increase the sintering time rather than the temperature to reach a better interface quality when using the MgAgSb/MgCuSb system.

Lastly, although a higher sintering pressure might enable the current flow field to expand more homogeneously due to a less porous electrode and a greater uniformity of the contact between MgCuSb and MgAgSb, 1step_575-30-85_ and 1step_575-30-200_ show similar contact resistance values within the measurement uncertainty, preventing any conclusion on the effect of sintering pressure on the electrical contact quality. However, it should be noted that these two samples were contacted using respectively a graphite die and a stainless-steel die, and that the cooling time of the steel die is 2.5 times larger than that of the graphite die. This matches the 2.5 times larger dyscrasite interface zone that can be observed for 1step_575-30-200_ (Figure 2), giving evidence again for the direct link between the thickness of the Ag_3_Sb precipitate zone and the sintering duration.

A close examination of the relationship between the thickness of the dyscrasite zone at the interface and the contact resistance of all samples indicates that the thickness of this phase does not appear to negatively impact the contact resistance. In fact, it seems to improve the contact, as evidenced when comparing the results from 1step_575-30-85_ and 1step_575-30-200_, as well as 1step_575-8-85_ and 1step_575-30-85_. This could be explained by the fact that the secondary phases present at the interface usually originate from the interdiffusion of elements between the electrode and the TE material, resulting in a strong bonding at the interface if the formed phases are stable. Consequently, if the electrical properties of the formed phases are comparable to those of the TE material, this can lead to a lower contact resistance. Our findings agree with those of Li et al. [67], who also found increasing Ag_3_Sb formation and decreasing contact resistance, both with increasing annealing time, and argued that the reduction in *r*_c_ could be explained by an increase in the charge carrier concentration of MgAgSb close to the interface. We have shown by microprobe measurements that the Seebeck coefficient decreases close to the interface, corresponding to an increase in carrier concentration, hence in line with the hypothesis presented there. However, even if a thicker Ag_3_Sb interface zone coincides with a better contact, it could present an issue in the long run, especially in thermoelectric modules subjected to extended duration of high hot side temperatures. Over time, this layer could contribute to progressive changes in the composition of the TE material, with the depth of alteration increasing with time. If the region of the thermoelectric material affected by this compositional shift is not negligible compared to the overall length, it will induce a change in the TE material properties, ultimately reducing the performance of the module (for an approach to estimate the impact of graded material properties on module performance, see [68]). In our case, further studies would need to be performed to evaluate the effect of the dyscrasite interface zone on the module performance over time.

This study highlights that the best sintering parameters are not necessarily those leading to the lowest contact resistance with the lowest standard deviation value (respectively indicating a better and more homogeneous contact quality), but that the impact on the microstructure and thus on the TE properties must also be taken into account. We identify here as optimum sintering parameters a temperature of 575 K to minimize copper diffusion, a duration of at least 30 min to allow a strong interdiffusion between the two materials and thus a better electrical contact, and a pressure of 85 MPa using a graphite die with a short cooling time.

Those parameters were thus used for the contacting of 2step_575-30-85_, which is the first reported MgAgSb sample successfully contacted using a two-step process. Indeed, this sample does not show any cracks or delamination at the interfaces (Figure 4) and exhibits a low contact resistance standing below 7 µΩ cm^2^, which represents less than 5% of the total bar resistance, assessing a good quality of the contact. We note that all samples show the formation of a dyscrasite interface zone at the MgAgSb/MgCuSb interface (Figures 2 and 4), whose thickness increases with increasing joining time and temperature. This is remarkable because Xie et al. [48] based their selection of MgCuSb as an electrode for MgAgSb on the prediction of a stable two-phase equilibrium between the two materials, indicating that no secondary phases should form. However, the employed quaternary phase diagrams were calculated at 0 K, while processing and application temperatures are obviously higher. This points out that the screening strategy employed in [48] might serve as a first indication but has limited predictive power for the actually relevant temperatures and cannot substitute experimental verification.

Figure 4b also shows the presence of a 6 μm large compacted MgCuSb layer in the electrode along the interface, as highlighted by the blue arrow, which is unexpected as a constant pressure of 85 MPa has been applied and should thus lead to a homogeneous electrode in terms of porosity. This layer is not visible at first sight for 1step_575-30-85_ contacted using the same parameters, although a non-porous region of around 6 μm from the interface can be distinguished by looking closely. The formation mechanism of this layer as well as its potential impact on the contact quality would need to be studied, for example with annealing to see its evolution with time.

**Figure 6.**
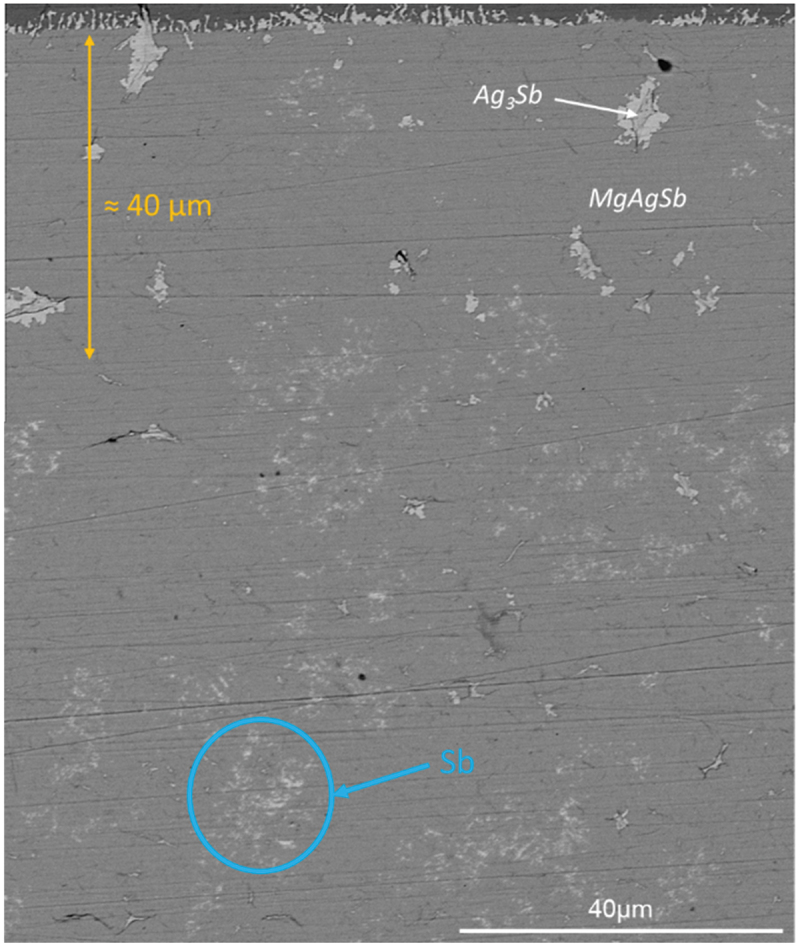
BSE-SEM view of the TE material close to the interface of sample 2step_575-30-85._ The top dark layer is the electrode (MgCusb). The white blurry precipitates circled in blue are elemental antimony.

The reduction of the Seebeck coefficient observed close to the interfaces in Figure 5 for 2step_575-30-85_ can in principle arise from the formation of secondary phases or from a change in the carrier concentration of the thermoelectric material, caused by a change in concentration of charged defects. The dyscrasite interface zone can be ruled out as origin as it is much thinner (≈3 µm as observed in Figure 4) than the diffusion length (90 µm calculated by fitting the Seebeck curve in Figure 5). A low magnification image of the TE material (Figure 6) indicates relatively large Ag_3_Sb particles present close to the interface (up to a depth of around 40 μm, as indicated by the yellow arrow) which cannot be found to the same extent deeper inside the sample. As Ag_3_Sb is metallic, it might cause the observed reduction of the Seebeck coefficient, but these larger Ag_3_Sb particles are only visible up to a distance of around 40 µm (Figure 6), smaller than the depth up to which a changed Seebeck coefficient can be observed (around 300 µm, as shown in Figure 5). It is also unlikely that several separate particles would cause such a gradual and homogeneous gradient in Seebeck coefficient rather than isolated spots showing low Seebeck coefficient values. Instead, we believe that this change in Seebeck coefficient is due to a change in the defect concentrations related to Cu and/or Ag diffusion, into or out of the TE material respectively, as evidenced by the exponential shape of the gradient. Indeed, it is known that silver vacancies (V_Ag_) are the dominant defects in Mg-rich MgAgSb [69], and that lattice diffusion is often governed by diffusion via vacancies [70], making this change of defect concentrations generally plausible. Ag diffusing out of MgAgSb towards the MgCuSb interface would facilitate the formation of Ag_3_Sb due to the excess of silver, and result in an increased number of V_Ag_, an acceptor defect, leading to an increasing charge carrier concentration towards the interface, in line with the observed decrease of the Seebeck coefficient [10]. On the other hand, Cu is known to be a fast diffuser in many materials [64] and we have observed proof for Cu diffusion across the interface (1step_625-8-85_, Figure 2c) into the TE material. Furthermore, Sui et al. [61] showed that Cu can be incorporated into MgAg_0.97_Sb_0.99_ with a solubility limit of around 0.7% of the Ag site, and observed a reduction of the Seebeck coefficient after nominally substituting Ag by Cu, presumably due to the formation of acceptor-type defects. As Cu and Ag are isovalent, this might be unexpected but it is possible that the presence of Cu changes the defect formation energies and hence the concentration of Ag-related defects. A similar effect is observed in the (Bi,Sb)Te system [71]. The amount of elements required to explain the observed changes in the Seebeck coefficient are too small to be detected by EDS, but both the change in carrier concentration due to the diffusion of an electrode species into the TE material as well as the diffusion of a TE element species into the electrode have been observed, for instance for Mg_2_(Si,Sn) contacted with several electrode materials [56,72], and could explain the results obtained in the case of the MgAgSb/MgCuSb interface.

## Conclusion and outlook

We have presented the first detailed study of contacting α-MgAgSb TE material using MgCuSb as the electrode, as well as the first ever reported two-sintering-step functionalization of MgAgSb TE material. All samples show morphologically good contacts, as no delamination or cracks can be seen at the interfaces and as the calculated electrical contact resistances are all below 10 μΩ cm^2^. The comparison of different samples contacted via a one-step process using different sintering durations, temperatures and pressures shows that an increase in sintering time and temperature leads to the formation of a thicker Ag_3_Sb precipitation zone along the interface, but also reduces the contact resistance, suggesting a stronger bonding of the two materials. However, increasing the sintering temperature also enhances the diffusion of Cu from the electrode, which would lead to a modification of the TE properties of MgAgSb material, and thus emphasizes the importance of finding a balance between optimizing the electrical and microstructural contact quality.

The two-step contacted sample exhibited a very low contact resistance below 7 µΩ cm^2^, representing less than 5% of the total resistance of the leg. However, we found that Ag and Cu are diffusing, from and to the TE material respectively, as evidenced by the formation of dyscrasite and the reduction of the Seebeck coefficient visible in MgAgSb close to the interface. The latter is caused by a change in the defect densities in MgAgSb, presumably an increase in silver vacancies. Future work should include annealing and cycling experiments to investigate the effect of thermal load on contact resistance, extension of the interdiffusion zone and further analysis of the observed interdiffusion processes and their effect on material and device performance.

Even though using MgCuSb as an electrode for MgAgSb leads to the formation of secondary phases at the interface (found here in contrast to previous reports), the successful contacting of MgAgSb through a two-step process marks a notable advancement in thermoelectric joining technology. This development enables improved control over TE leg length, bringing the accomplishment of practical applications within closer reach.

## Supplementary Material

Supplemental Material
