# Supplementary material for: MgCuSb as a suitable electrode for contacting pre-compacted pellets of MgAgSb thermoelectric material
Source: Sci Technol Adv Mater. 2025 May 27;26(1):2506982. doi: 10.1080/14686996.2025.2506982 (PMC12160332; doi:10.1080/14686996.2025.2506982)
Supplement: Supplemental Material [file TSTA_A_2506982_SM1399.docx]

**Supplementary information**

*Figure S1: Comparison of XRD patterns for the one- and two-step ball milling synthesis of MgCuSb. The right graph corresponds to a zoom-in from the pink section of the left graph*


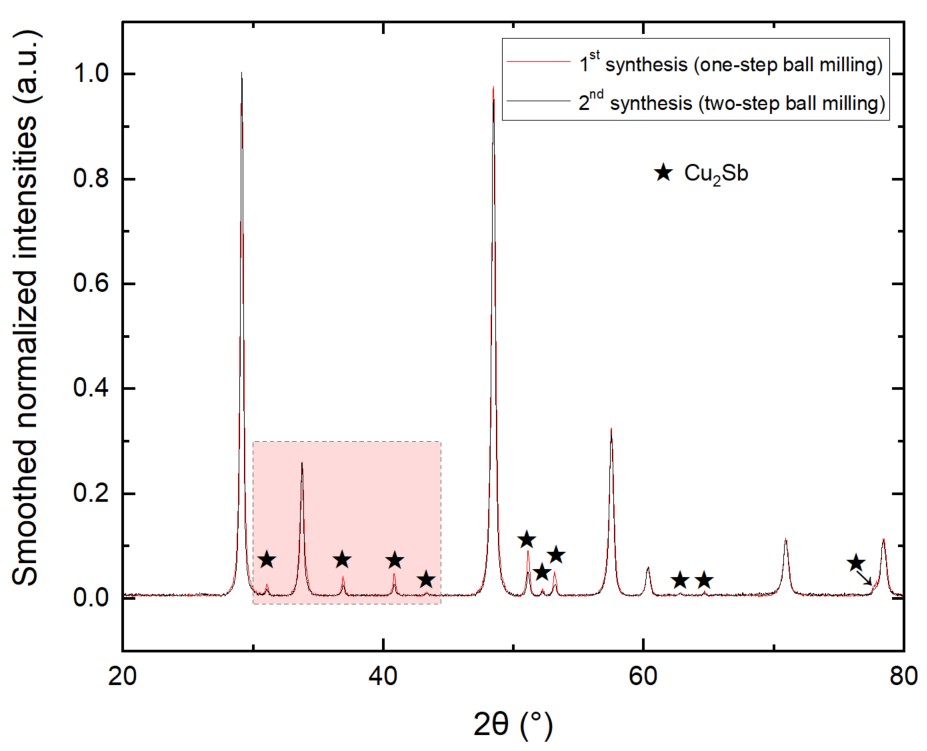

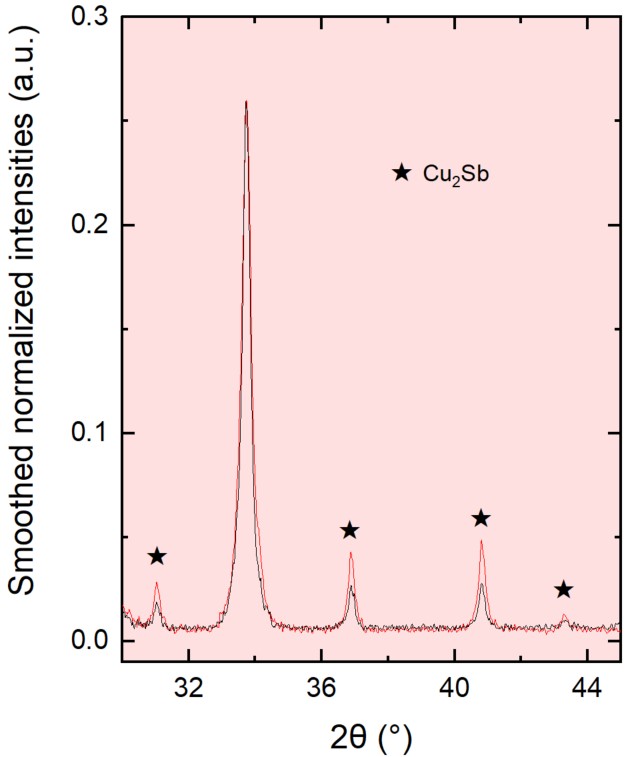


One can clearly see that the intensity of the Cu_2_Sb peaks reduces between the first and second synthesis, in line with the decrease from 10 wt. % to 6 wt. % of the Cu_2_Sb content (Table S1 & Table S2).

*Table S1: Rietveld refinement values of the first MgCuSb synthesis (one-step ball milling)*

|  | **R-values** | |  | |
| --- | --- | --- | --- | --- |
| *R-weighted pattern (Rwp)* | *R-Bragg MgCuSb* | *R-Bragg Cu_2_Sb* |  | *Goodness of fit (GOF)* |
| 12 | 1.2 | 6.4 |  | 1.8 |
|  | **Quantitative analysis** | |  | |
| Main phase | MgCuSb |  | 89.6 wt. % | |
| Secondary phase | Cu_2_Sb |  | **10.4 wt. %** | |

*Table S2: Rietveld refinement values of the second MgCuSb synthesis (two-step ball milling)*

|  | **R-values** | |  | |
| --- | --- | --- | --- | --- |
| *R-weighted pattern (Rwp)* | *R-Bragg MgCuSb* | *R-Bragg Cu_2_Sb* |  | *Goodness of fit (GOF)* |
| 10.5 | 1.1 | 5.3 |  | 1.7 |
|  | **Quantitative analysis** | |  | |
| Main phase | MgCuSb |  | 93.92 wt. % | |
| Secondary phase | Cu_2_Sb |  | **6.08 wt. %** | |

*Figure S2: Backscattered electron (BSE)-SEM images of the two MgCuSb pellets obtained from a) the one-ball-milling-step synthesis route and b) the two-ball-milling-step synthesis route.*

a)

b)


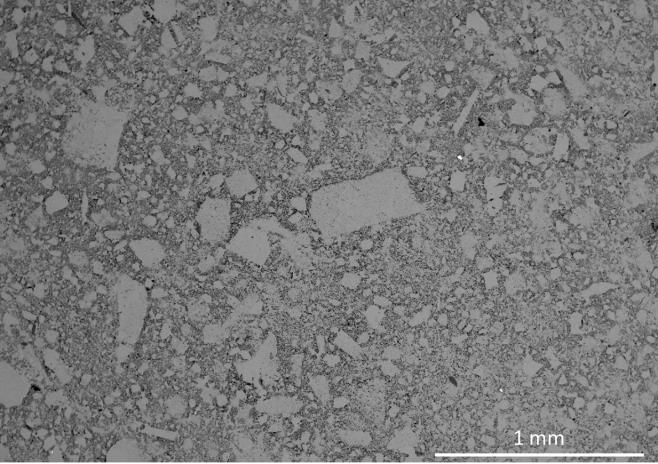

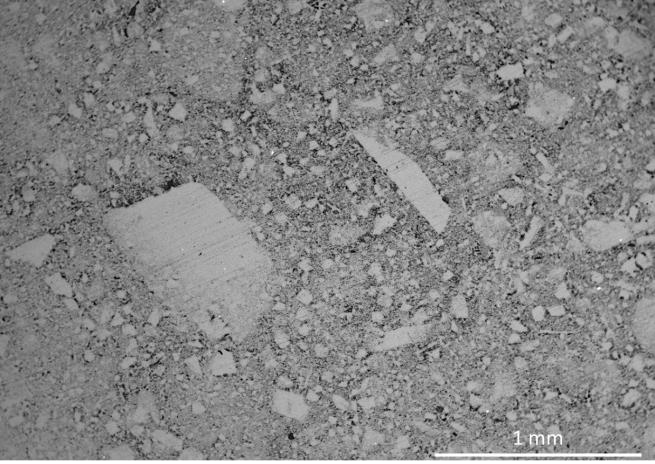


*Table S3: Properties measured on the MgCuSb pellets obtained from the two different synthesis routes*

| **Property/ characterization** | **One-HEBM-step synthesis route** | **Two-HEBM-step synthesis route** |
| --- | --- | --- |
| **Density (g cm^-3^)** | 4.5 | 5.3 |
| **Relative density (%) *** | 78.5 | 92.5 |
| **Seebeck (μV K^-1^)** | 17.5 | 17 |
| **Electrical conductivity**  **(S cm^-1^)** | 4000 | 7000 |
| **Thermal conductivity**  **(W m-1 K-1)** | 7.4 | 7.3 |
| **Cu_2_Sb content (wt. %)** | 10.4 | 6.4 |

*The theoretical density of MgCuSb is 5.73 g cm^-3^.

*3: Explanation of the MATLAB program used for contact resistance calculations*


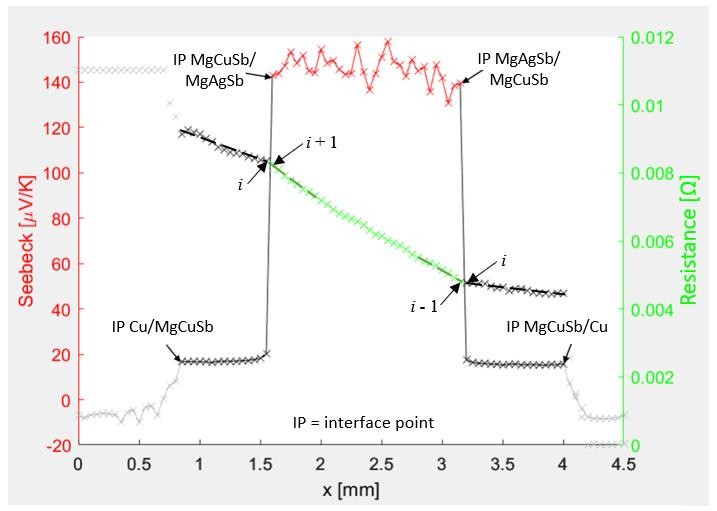


*Seebeck/Resistance values versus x-position. The grey lines represent the values in the sample holder, the black lines correspond to the electrodes, and the red and green lines are respectively the values of the Seebeck coefficient and the resistance in the TE material. The points defined as i and i*

*± 1 are used to calculate the contact resistances. The black dashed lines correspond to the fits in the electrodes and the green ones to the fits in the TE material. IP indicates an interface position.*

The MATLAB program developed to automatize the calculation of the specific electrical contact resistances works as follow:

- First of all, the geometry parameters must but input by the user, and the Seebeck coefficient and resistance values for all x and y-positions on the sample should be imported by the code.

- The position of the interfaces between the electrode and the TE material, as well as between the sample holder and the electrode, are then determined on both sides using the abrupt changes in Seebeck value. The Seebeck/Resistance versus x-position graphs are displayed for all y-values (see figure above), which enables to check if the interface points (IP on the figure) were correctly determined.

- A linear fitting of the resistance curves *R(x)* is then done in both electrodes (see black dashed lines in previous figure) using the previously determined interface points as limits, and this for all the graphs *i.e.* for all y-values. The same kind of fitting is also done in two portions of the TE material, left and right sides close to the interfaces with the electrode, as represented by the green dashed lines. Doing this partial fitting instead of a global fitting on all the TE material enables to reduce uncertainties linked to the curvature of *R(x)*. Indeed, this latter is a straight line in all the TE range, except close to the interfaces where it can be slightly bent. The linear correlation coefficients are calculated for each segment and will then be used as conditions to determine if the fitting is accurate enough to use its values. Indeed, if the value of the correlation coefficient is lower than 0.8 either on the left or right side, it means that the difference between the measured and fitted resistance values is too large to consider the corresponding in the rest of the analysis.

# μm

• The contact resistance is calculated for the remaining graphs, as explained here for the left side. First of all, several points need to be defined, as showed in the explanatory figure:

o 𝑅_𝑒𝑙𝑒𝑐,𝑖_ : resistance value given by the linear fitting of the electrode at the last point in the electrode o 𝑅_𝑇𝐸,𝑖_ : resistance value given by the linear fitting of the TE material at the last point in the electrode o 𝑅_𝑒𝑙𝑒𝑐,𝑖+1_ : resistance value given by the linear fitting of the electrode at the first point in the TE material o 𝑅_𝑇𝐸,𝑖+1_ : resistance value given by the linear fitting of the TE material at the first point in the TE material Then, the contact resistance is given by the following equation:

(𝑅𝑒𝑙𝑒𝑐,𝑖 − 𝑅𝑇𝐸,𝑖) + (𝑅𝑒𝑙𝑒𝑐,𝑖+1 − 𝑅𝑇𝐸,𝑖+1)

𝑅𝑐,𝑙𝑒𝑓𝑡 =

2

On the right side, subscripts *i* and *i*-1 are used to represent respectively the last point in the electrode and the first point in the TE material. The contact resistance is then calculated using the next equation:

(𝑅_𝑇𝐸_,𝑖−1 − 𝑅_𝑒𝑙𝑒𝑐_,𝑖) + (𝑅_𝑇𝐸_,𝑖 − 𝑅_𝑒𝑙𝑒𝑐_,𝑖)

𝑅𝑐,𝑟𝑖𝑔ℎ𝑡 =

2

Once the contact resistances are obtained for both left and right interfaces, the specific electrical contact resistance can be calculated by multiplying the obtained contact resistance by the cross-section that has been input at the beginning, as shown in equation (3).

- The electrical conductivity is also calculated using the slope of the resistance curve ^(𝛥𝑅^ < 0) in the TE material

𝛥𝐿

range:

𝐿 1


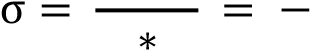
 𝑅 𝐴 𝑠𝑙𝑜𝑝𝑒 ∗ 𝐴

- Finally, the average values of left, right and global specific contact resistance, as well as the *n* ratio (defined in equation (4)) are displayed in the console, all with their respective error values. Those errors are calculated using the standard deviation of the respective parameter along the y-range. It should be noted that, for the calculation of *R_total_* in the *n* ratio, the electrical conductivity value after annealing in the high temperature Seebeck and Sigma measurement device is employed, even if the sample has not been annealed before the PSM measurement. Indeed, this is the most representative value for the module characterization case as the first heating/cooling cycle will act like an annealing step.

This method presents several advantages such as its rapid execution and its reproducibility as it is independent of the user’s analysis method. Moreover, using linear fittings enables to get more accurate data by averaging the statistical noise of the measurement values, and also allows the extrapolation to the interface point. However, irregularities and especially non-linearities in the resistance curves can induce errors due to the linear fitting that cand lead to negative contact resistance values for some of the y-positions and to an underestimation of the total contact resistance. However, this only occurs in the case of small resistance differences at the interface, *i.e.* low contact resistances. Consequently, while negative contact resistances are an indication for scattered data or non-linear potential curves, they are also an indication for contacts with excellent electrical properties here.

*4: BSE SEM low magnification images of a) 1step_575-8-85_, b) 1step_625-8-85_ and c) 1step_575-30-200_*


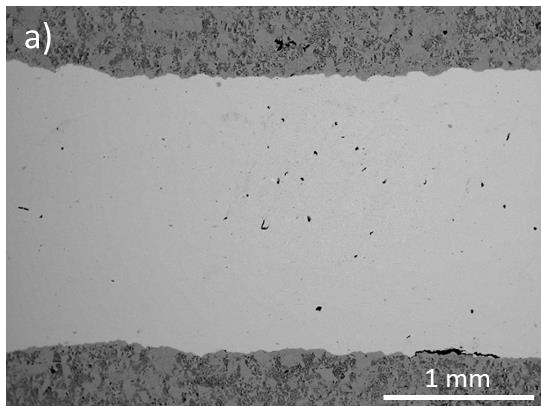

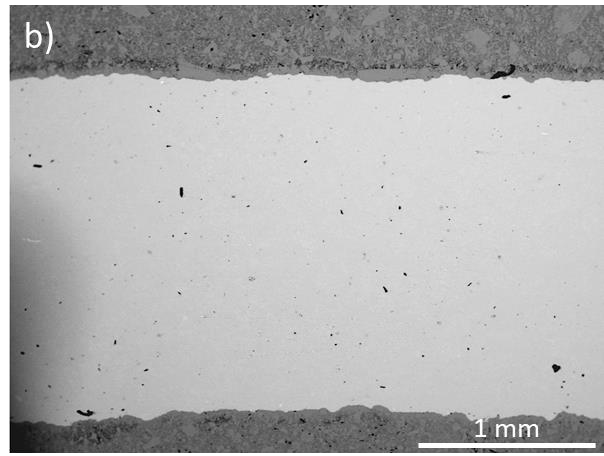


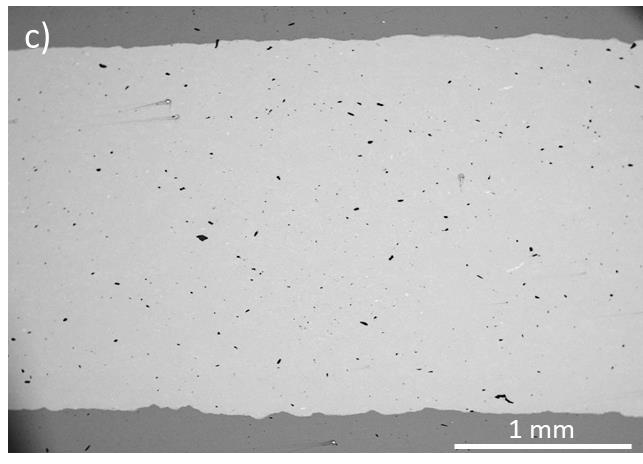


*5: BSE SEM image of a non-contacted MgAgSb sample showing the presence of secondary phases (Ag_3_Sb and Sb)*


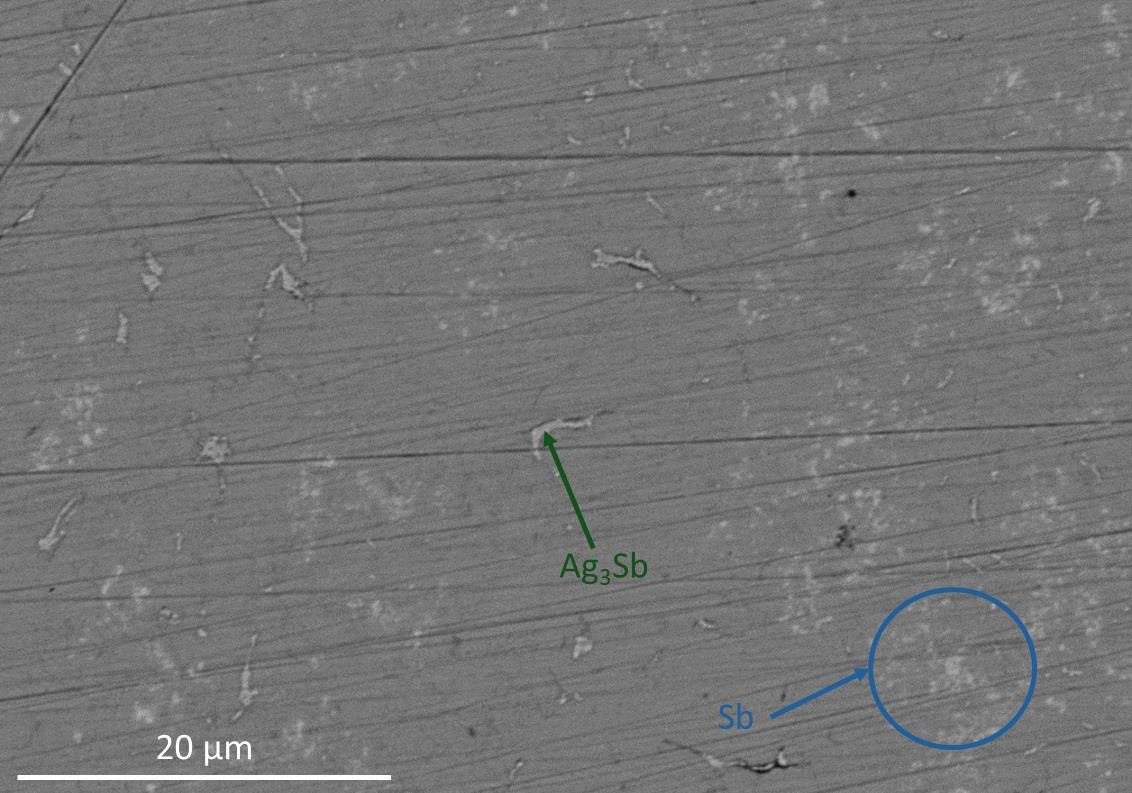


*6: EDS SEM analysis of a non-contacted MgCuSb pellet made with the same powder as that used for the contacting of 1step_625-8-85_*


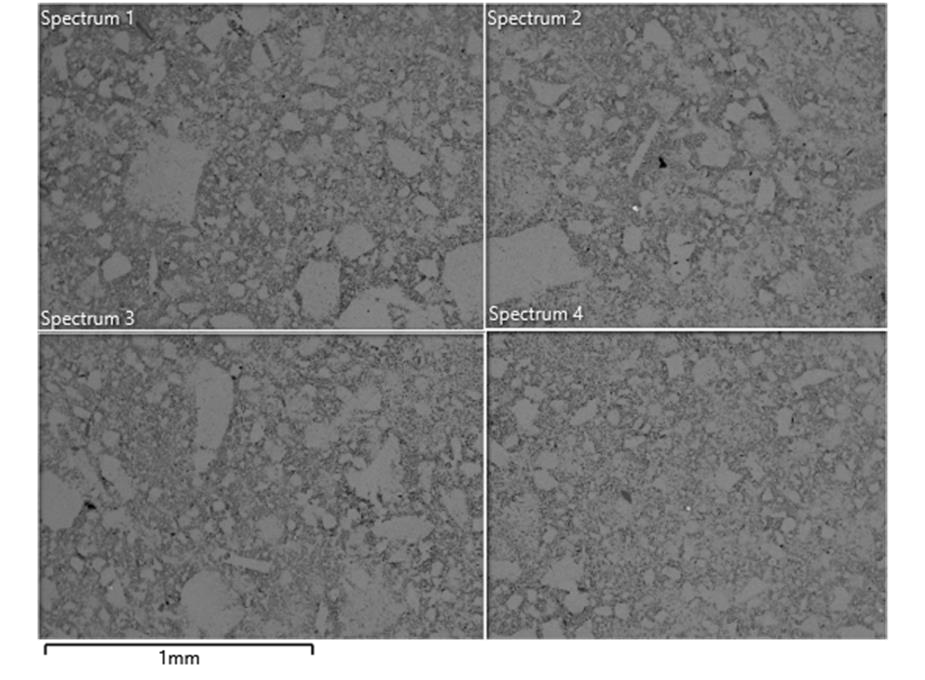


| **Spectrum** | **Mg (at. %)** | **Cu (at. %)** | **Sb (at. %)** |
| --- | --- | --- | --- |
| 1 | 32.1 | 35.4 | 32.4 |
| 2 | 32 | 35.3 | 32.8 |
| 3 | 32.4 | 34.7 | 32.9 |
| 4 | 32 | 35.2 | 32.9 |
| **Global** | **32.1** | **35.2** | **32.9** |

This non-contacted sample contains 35.2 at. % of Cu, whereas the electrode of sample 1step_625-8-85_ is composed of 29.6 at.

% Cu (see point 4 in Figure 2-c and Table 2), which shows that contacting induces diffusion of copper from the electrode

to the TE material. The measured values for Mg and Sb are comparable for both samples.

*7: PSM scans of a) 1step575-8-85, b) 1step625-8-85, and c) 1step575-30-200*


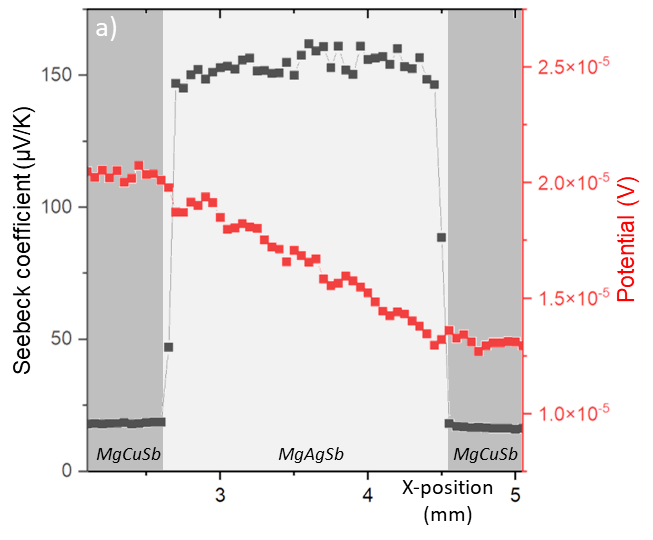


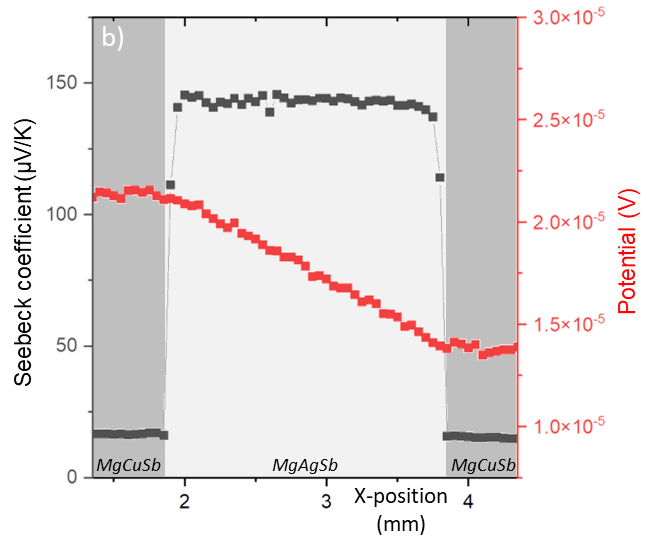


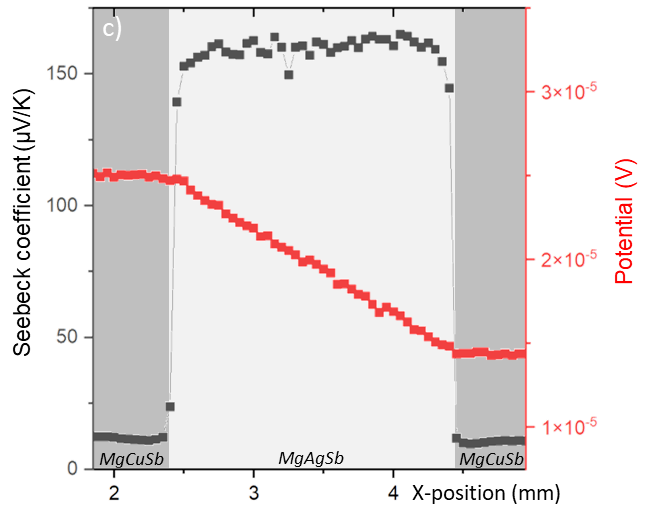


*8: Zoom on the top interface of 2step_575-30-85_ and corresponding* *EDX point analysis.*


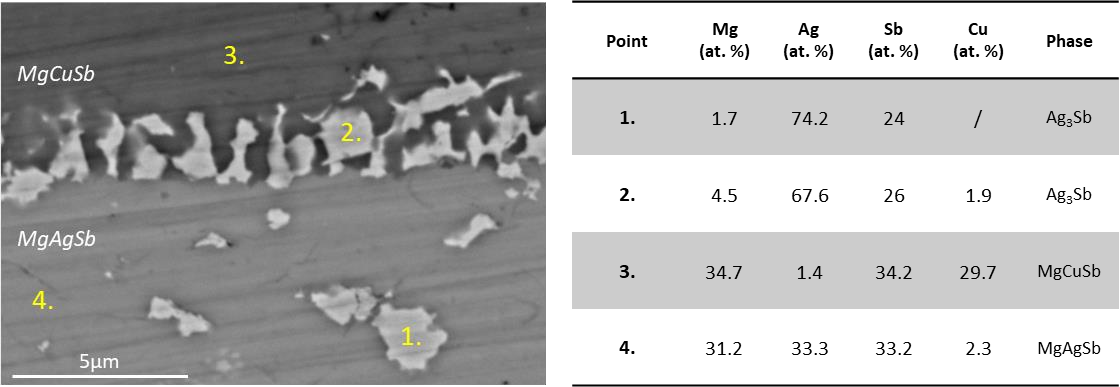


This EDX analysis evidences the presence of dyscrasite (Ag_3_Sb) at the interface, as well as the fact that the compacted layer highlighted by a blue arrow in Figure 4 is composed of MgCuSb.
